# A New Light on Florence Nightingale

**Published:** 1921-04-09

**Authors:** 


### A New Light on Florence Nightingale.

Books are necessarily a better guide than the newspaper to the influence which a person exerts on his or her own time, for the books are not written till the generation which they record are over, when we find that history was made by the handful of people who knew their own minds and so became centres of influence. An illustration is supplied by the growing number of books which contain mention of Florence Nightingale. The latest is Mr. Shane Leslie's "Henry Edward Manning" (Burns, Oates and Washbourne, Ltd., 25s. net). Written as a counterblast to Purcell's life of the Cardinal, which was "potted" so amusingly by Mr. Lytton Strachey in 1918, it contains a chapter on Miss Nightingale and the Cardinal. She was one of the people whom Manning did his best to "convert," but his quiver, full of remarkable names, never gained hers, the most eminent of his penitents. At first she poured out many of her thoughts to him, but they came together when he seconded her efforts to arrange nursing for the wounded in the Crimea. Florence Nightingale and Miss Stanley were allies in this task, and while Manning aided them he sought their conversion unremittingly. The pair first became intimate when Manning was in Rome in 1847. His encouragement must have been very cheering, for in both the organising quality was in excess of the apprehensive, and to be "practical" was coming into fashion.

Manning made some shrewd criticisms of her. Mr. Shane Leslie quotes this: "Empirically but not scientifically, I believe in her; she has no more fervent disciple than I. I believe in her as the early Chaldeans believed in the return of eclipses, which they could ascertain by observation but could not account for." Her criticism of Manning was this: His reasons for entering the Church of Rome, she said, "were of the Oxford historical school"; she "preferred the course of the mathematical and Cambridge men, who examine each doctrine 'whether they can believe in it' rather than 'whether they can believe in her.'" How well we know her by her argument here!

After a visit to Belfast in 1852 she wrote, "Imagine a new commercial, Orange, Presbyterian town, a cross between Geneva and Manchester, inhabited by that anomalous animal an Irish Protestant, with infirmaries, poorhouses, all on the model of London." Such correspondence could not in the long run have united the two; but the call for action in the Crimean War sealed their mutual respect. It "inspired Manning to find chaplains and Miss Nightingale nurses for the British Army." In the end Manning found sisters also from the convents, so compelling was Miss Nightingale's influence, and so eager was he to see her at the head of the great work. Into the debatable question of jurisdiction between Florence Nightingale and the superiors of the sisters we cannot enter. Manning helped her loyally, and the expedition was saved. The book is a supplement to Purcell, but will not replace it. For it is somewhat scrappy, a compilation rather than a life, without a real texture of style, and with the suggestion, no doubt unconscious, of having been written to order. But it should not be missed, even though, like every book referring to Miss Nightingale, it leads the reader back to Sir Edward Cook's monumental biography. If one may say so without offence, the subtlest attempt to reveal the secret of Miss Nightingale is still the article published in The Hospital of October 19, 1918, under the title of "The Nightingale Legend," for that was based upon an intimate knowledge of her own books, where she reveals herself as one of the great, if incomplete, mystics of the nineteenth century.

